# Genetic basis and spatial distribution of glucose-6-phosphate dehydrogenase deficiency in ecuadorian ethnic groups: a malaria perspective

**DOI:** 10.1186/s12936-023-04716-x

**Published:** 2023-09-26

**Authors:** Sebastián Atarihuana, Jennifer Gallardo-Condor, Andrés López-Cortés, Karina Jimenes-Vargas, Germán Burgos, Ana Karina-Zambrano, Rodrigo Flores-Espinoza, Marco Coral, Alejandro Cabrera-Andrade

**Affiliations:** 1 Facultad de Ingeniería y Ciencias Aplicadas, Universidad de Las Américas, Quito, Ecuador; 2 Cancer Research Group (CRG), Faculty of Medicine, Universidad de Las Américas, Quito, Ecuador; 3 Latin American Network for the Implementation and Validation of Clinical Pharmacogenomics Guidelines (RELIVAF-CYTED), Madrid, Spain; 4 Grupo de Bio-Quimioinformática, Universidad de Las Américas, Quito, Ecuador; 5 Department of Computer Science and Information Technologies, Computer Science Faculty, CITIC, RNASA Group, University of A Coruña, A Coruña, Spain; 6 One Health Research Group, Facultad de Medicina, Universidad de las Américas, Quito, Ecuador; 7 Grupo de Medicina Xenómica, Instituto de Ciencias Forenses, Universidad de Santiago de Compostela, A Coruña, Spain; 8 Centro de Investigación Genética y Genómica, Facultad de Ciencias de la Salud Eugenio Espejo, Universidad UTE, Quito, Ecuador; 9 Laboratório de Diagnóstico por DNA (LDD), Universidade do Estado do Rio de Janeiro, Rio de Janeiro, Brazil; 10 Carrera de Medicina Veterinaria, Facultad de Ciencias de la Salud, Universidad de Las Américas, Quito, Ecuador; 11 Escuela de Enfermería, Facultad de Ciencias de la Salud, Universidad de Las Américas, Quito, Ecuador

**Keywords:** Glucose-6-phosphate dehydrogenase, G6PD deficiency, Single nucleotide variants, Malaria clusters, Ecuador, Afro-Ecuadorians, Pharmacogenetics

## Abstract

**Background:**

Glucose-6-phosphate dehydrogenase deficiency (G6PDd) is an X-linked disorder affecting over 400 million people worldwide. Individuals with molecular variants associated with reduced enzymatic activity are susceptible to oxidative stress in red blood cells, thereby increasing the risk of pathophysiological conditions and toxicity to anti-malarial treatments. Globally, the prevalence of G6PDd varies among populations. Accordingly, this study aims to characterize G6PDd distribution within the Ecuadorian population and to describe the spatial distribution of reported malaria cases.

**Methods:**

Molecular variants associated with G6PDd were genotyped in 581 individuals from Afro-Ecuadorian, Indigenous, Mestizo, and Montubio ethnic groups. Additionally, spatial analysis was conducted to identify significant malaria clusters with high incidence rates across Ecuador, using data collected from 2010 to 2021.

**Results:**

The A- c.202G > A and A- c.968T > C variants underpin the genetic basis of G6PDd in the studied population. The overall prevalence of G6PDd was 4.6% in the entire population. However, this frequency increased to 19.2% among Afro-Ecuadorian people. Spatial analysis revealed 12 malaria clusters, primarily located in the north of the country and its Amazon region, with relative risks of infection of 2.02 to 87.88.

**Conclusions:**

The findings of this study hold significant implications for public health interventions, treatment strategies, and targeted efforts to mitigate the burden of malaria in Ecuador. The high prevalence of G6PDd among Afro-Ecuadorian groups in the northern endemic areas necessitates the development of comprehensive malaria eradication strategies tailored to this geographical region.

**Supplementary Information:**

The online version contains supplementary material available at 10.1186/s12936-023-04716-x.

## Background

Glucose-6-phosphate dehydrogenase (G6PD) is an evolutionary conserved enzyme associated with the pentose phosphate pathway. As an oxidoreductase, it generates reduced nicotinamide adenine dinucleotide phosphate (NADPH) as a byproduct of glucose oxidation [1]. This metabolite plays an essential role in cellular reactions against oxidative stress, as NADPH regenerates reduced glutathione, a potent antioxidant that protects the cell from reactive oxygen species like hydrogen peroxide [2].

G6PD deficiency (G6PDd) is an X-linked disorder characterized by reduced enzymatic activity. This reduction primarily stems from molecular variants, which adversely affect the enzyme’s stability. Over 200 mutations have been identified in the *G6PD* gene to date, with most of them being single nucleotide variants (SNVs) [3]. The World Health Organization classifies each allelic variant of *G6PD* into one of five levels, considering the percentage of reduction in enzymatic activity [4]. While the class I Harilaou c.648G (rs137852319) variant represents the most severe deficiency mutation, classes IV and V represent the least severe. The class II Mediterranean c.563T (rs5030868) variant leads to a residual G6PD activity of less than 10%; carriers of this deficiency are more likely to develop diseases triggered by intrinsic or extrinsic oxidative stressors [5]. Class III variants c.202 A (rs1050828) and c.968 C (rs76723693) retain a range of activity from 10 to 60%. Since these latter classes decrease G6PD activity, their presence is associated with acute hemolytic anemia. Class IV c.376G (rs1050829) is related to enzymatic activity from 60 to 100%, which is considered normal enzyme activity [6].

Reduced activity of G6PD affects NAPDH homeostasis, particularly in red blood cells, leading to varying conditions based on enzyme activity levels [7]. Individuals might remain asymptomatic until exposed to oxidative stressors like high consumption of fava beans (favism). A significant activity reduction can cause chronic non-sphaerocytic haemolytic anaemia, presenting symptoms like jaundice and splenomegaly [8]. In children, G6PDd-related haemolytic anaemia symptoms include fatigue and irritability [9], while in adults, manifestations vary based on genetic variations and deficiency severity. Typically, treating haemolytic anaemia involves removing the triggering factor, like certain drugs or fava beans [10].

G6PDd also has significant implications in the context of malaria, particularly in terms of treatment efficacy and the development of toxicity-associated conditions. The recommended treatment drug for eliminating *Plasmodium vivax* hypnozoites and *Plasmodium falciparum* gametocytes is the 8-aminoquinoline drug family, including primaquine and tafenoquine. G6PDd individuals generate toxicity upon administration of these drugs, probably due to an imbalance in the redox reactions within blood cells, favouring the formation of highly reactive oxidized metabolites of primaquine [11]. Patients receiving treatment for *P. vivax* malaria are at a higher risk of developing haemolytic anaemia because of the increased dose of primaquine they receive (0.25–0.5 mg over a 14-day treatment period), compared to those with *P. falciparum* malaria (a single dose of 0.25 mg on the first day of treatment) [12]. While higher doses of primaquine effectively clear primary infections and prevent relapse in *P. vivax* patients compared to lower doses, there are inherent risks due to factors the unknown G6PD levels in many patients [13, 14].

The diversity of genetic factors contributing to G6PDd is closely linked to ethnogeography. The reduced function Mediterranean c.563T variant is prevalent in the Middle Eastern and Southern Italy. Similarly, the Mediterranean together with the Seattle c.844 C (rs137852318) variants are prevalent for European groups, with allelic frequencies for the minor allele (MAFs) of 0.07% and 0.11% respectively [15, 16]. In Asia, although the Mahidol c.487 A (rs137852314) variant is the best-characterized and is considered predominant across Myanmar and Thailand [17, 18], additional variants are described in different regions of the continent. Thus, the Canton c.1376T (rs72554665) and Kaiping c.1388 A (rs72554664) variants are frequent in East Asia (MAF of 1.1% and 0.7%, respectively), while the Mediterranean (1.74%) and Kerala c.949 A (rs137852339) variants are frequent in the south, with frequencies of 1.74% and 1.14%, respectively [19]. The African A-haplotype, formed by the combination of the c.202 A/c.376G (rs1050828/rs1050829) or c.968 C/c.376G (rs76723693/rs1050829) variants, are majority for African populations (MAFs = of 11.6–0.24%, and 0.5% respectively) and for Latin American populations (MAFs of 0.4% and 0.08% respectively [15, 20, 21]. These molecular markers largely describe G6PD deficiency for these population groups.

In Latin America, malaria is considered endemic, especially in coastal and Amazonian areas, where both *P. vivax* and *P. falciparum* are prevalent [22, 23]. Even though G6PDd frequencies in Latin America are relatively low, with mean frequencies of ≤ 1% observed in Mexico, Guatemala, Peru, Bolivia, Uruguay, Chile, and Argentina, the highest prevalence is found in the Caribbean islands, French Guiana, Suriname, Guyana, northwestern Venezuela, and the Pacific coastal regions of Colombia, where the estimated prevalence exceeds 10%. In the Amazon region, prevalence estimates range from 4% in southern areas to 10% along the border with Guyana [24]. Although malaria has been nearly eradicated in Ecuador, it is still considered endemic, with occasional outbreaks caused by both parasites (*P. vivax* and *P. falciparum*) [25, 26]. Previous studies conducted on Afro-descendant groups in Ecuador indicate G6PDd rates of approximately 12–14% [27, 28]. However, these studies were limited to specific geographic regions, involved a small number of participants, and did not apply an accurate genetic screening strategy covering the main pharmacogenetic variants associated with G6PDd. Hence, the objective of this study was to analyse the molecular variants of *G6PD* associated with reduced enzymatic activity in various ethnic groups across Ecuador through a genetic screening approach employing Sanger sequencing, targeting multiple coding region sequences of the *G6PD* and with a specific focus on prevalent variants such as African A- c.202 A, A- c.968 C, and Mediterranean c.563T. The aim is also to provide a spatial distribution of reported malaria infections thus far. Describing the allelic and genotypic frequencies of *G6PD* molecular variants associated with decreased enzymatic activity will not only enrich the genetic characterization of this diverse Latin American population, but will also aid in the development of genetic screening strategies to identify individuals with G6PDd. The findings of this study will have implications for improving anti-malarial drug therapy in the Ecuadorian population, particularly for those groups residing in areas where malaria is endemic.

## Methods

### Sample collection

The sampling process was conducted during the period 2017 to 2018, and a total of 292 healthy men and 289 healthy women of Ecuadorian nationality, unrelated and of legal age, were included in the study. On the contrary, people who did not have Ecuadorian nationality, minors, and people sharing first-generation ancestors, were excluded from the study. To assess genetic diversity in current Ecuadorian populations, participants from the 3 main regions of Ecuador were considered: Coast (CO), Andes (HG), and Amazonia (AZ). In addition, ethnic self-determination was considered, and the sampled individuals were classified into 4 ethnic groups: Afro-Ecuadorian (AFE), Indigenous (IND), Mestizo (MEZ), and Montubio (MON). The MEZ group was sampled in the provinces of: El Oro, Esmeraldas, Guayas, Los Ríos, Manabí, Santa Elena, and Santo Domingo (CO); Azuay, Bolívar, Carchi, Cañar, Chimborazo, Cotopaxi, Imbabura, Loja, Pichincha, and Tungurahua (HG); and Morona Santiago, Napo, Pastaza, and Sucumbíos (AZ). The Montubio group was limited to the provinces of Los Ríos and Manabí (CO region). IND groups were selected from the provinces of: Chimborazo, Cotopaxi, and Imbabura (HG region); Morona Santiago, Napo, Pastaza, Orellana, and Zamora Chinchipe (AZ region); and Santo Domingo (CO region). Finally, the AFE groups were included from the provinces of: Esmeraldas province (CO region); and Imbabura and Carchi (HG region).

Each participant signed an informed consent form for population genetic studies. The applied experimental methodology was approved by the Ethical Committee in Human Research from the Universidad de las Américas, with registration number CEISH-UDLA 2017 - 0301.

### DNA extraction

Genomic DNA was extracted from blood samples using the salting-out method [29]. DNA was quantified using a NanoDrop ND-1000 Spectrophotometer (NanoDrop Technologies, Willmington, DE, USA) at 260 nm. The quality of the genetic material was evaluated by the absorbance values at 280 and 230 nm, and by electrophoresis in 1% agarose gel.

### Genotyping of G6PD SNVs and statistical analysis

For the design of the primers, 4 classes of SNVs related to a decreased enzymatic activity of G6PD were considered, and the Primer-BLAST tool was used (https://www.ncbi.nlm.nih.gov/tools/primer-blast/), reference sequence NG_009015.2 (GRCh38.p13). The amplified regions included the class I Harilaou c.648T > G (rs137852319) variant, the class II Mediterranean c.563 C > T (rs5030868) variant, the class III A- c.202G > A (rs1050828) and A- c. 968T > C variants, and the class IV SNV A- c.376 A > G (rs1050829). In addition, the SNVs c.1365-13 C > T (rs2071429) and c.1431 C > T (rs77214077) described as likely benign were screened.

PCR reaction was performed with the GoTaq® Green Master Mix (Promega, Madison, Wisconsin, USA) in a final volume of 15 µl, with 0.3 µMol of each primer and ~ 20 ng of genomic DNA. Amplified products were tested by electrophoresis in a 2% agarose gel and purified using exonuclease I (Exo I) and alkaline phosphatase (FastAP™) (Thermo Scientific™) according to the manufacturer’s standard protocol.

Sequencing reactions were performed using the BigDye® Terminator v3.1 Cycle Sequencing Kit (Applied Biosystems, Austin, TX, USA), following the manufacturer’s instructions. Within the reaction, a final concentration of 1 µM of primer was used in a volume of 10 µl. Next, amplicons were purified using Sephadex G-50 Fine (GE Healthcare) and run in an ABI 3130 Genetic Analyzer 142 (Applied Biosystems, Foster City, CA, USA). Finally, the sequences were aligned and analysed in the software Geneious Prime, version 2022.1.1.

Population genetic parameters, such as allele frequencies, gene diversity, Hardy-Weinberg Equilibrium (HWE), pairwise genetic distances (F_ST_), and analysis of molecular variance (AMOVA), were calculated using Arlequin software v.3.5.2.2. HWE analysis was performed only for females, and LD analysis in males. The significance level of 0.05 was adjusted by applying Bonferroni’s correction for multiple tests.

From the F_ST_ indices, a cluster analysis was applied to evaluate the degree of association between the population groups studied, using the Euclidean distance and Ward’s method for grouping. For the visualization of these data, heatmaps and dendrograms were developed in RStudio.

### Spatial analysis and statistical methods

A spatial cluster analysis was conducted to identify significant malaria clusters with high incidence rates across Ecuador, following the methodology described in previous studies [30, 31]. The analysis used the National Archive of Data and Statistical Metadata of the Ecuadorian National Institute of Statistics and Censuses (INEC) [32]. This study encompassed all documented malaria cases, categorized by canton and ICD-10 identification code, covering the time frame from 2010 to 2021. Incidence rates were adjusted based on population projections by year and sex, using exact 95% Poisson confidence intervals. These rates were reported in terms of absolute numbers of new cases and relative rates per 100,000 members of the population.

By comparing the reported cases with the spatial coordinates of each canton, the occurrence of cases in different areas was assessed using a Poisson distribution. Spatial clustering was evaluated by comparing the incidence rates in specific areas to the expected rate if cases were randomly distributed. The statistical significance of spatial clusters was determined using a likelihood test, with 999 Monte Carlo simulations employed to calculate the associated p-values. Any spatial clusters with a p-value below 0.05 were considered statistically significant. Additionally, the Gini coefficient [33] was used to make further selections among the significant clusters.

The identification of statistically significant spatial clusters was carried out using SATSCAN (version 10.1.2). Spatial analyses were performed using QGIS software (version 3.32.0), while the calculation of incidence rates was performed using the Epitools package, R version 4.1.3.

## Results

### G6PD genotyping

Five regions in *G6PD* were amplified to genotype previously described variants using the primers described in Additional file 1: Table S1. In the screened Ecuadorian population, the presence of class III and class IV variants is observed, while no polymorphic genotypes are recorded for class I Harilaou (c.648T > G) or class II Mediterranean (c.563 C > T) variants (Additional file 1: Table S2).

**Fig. 1:**
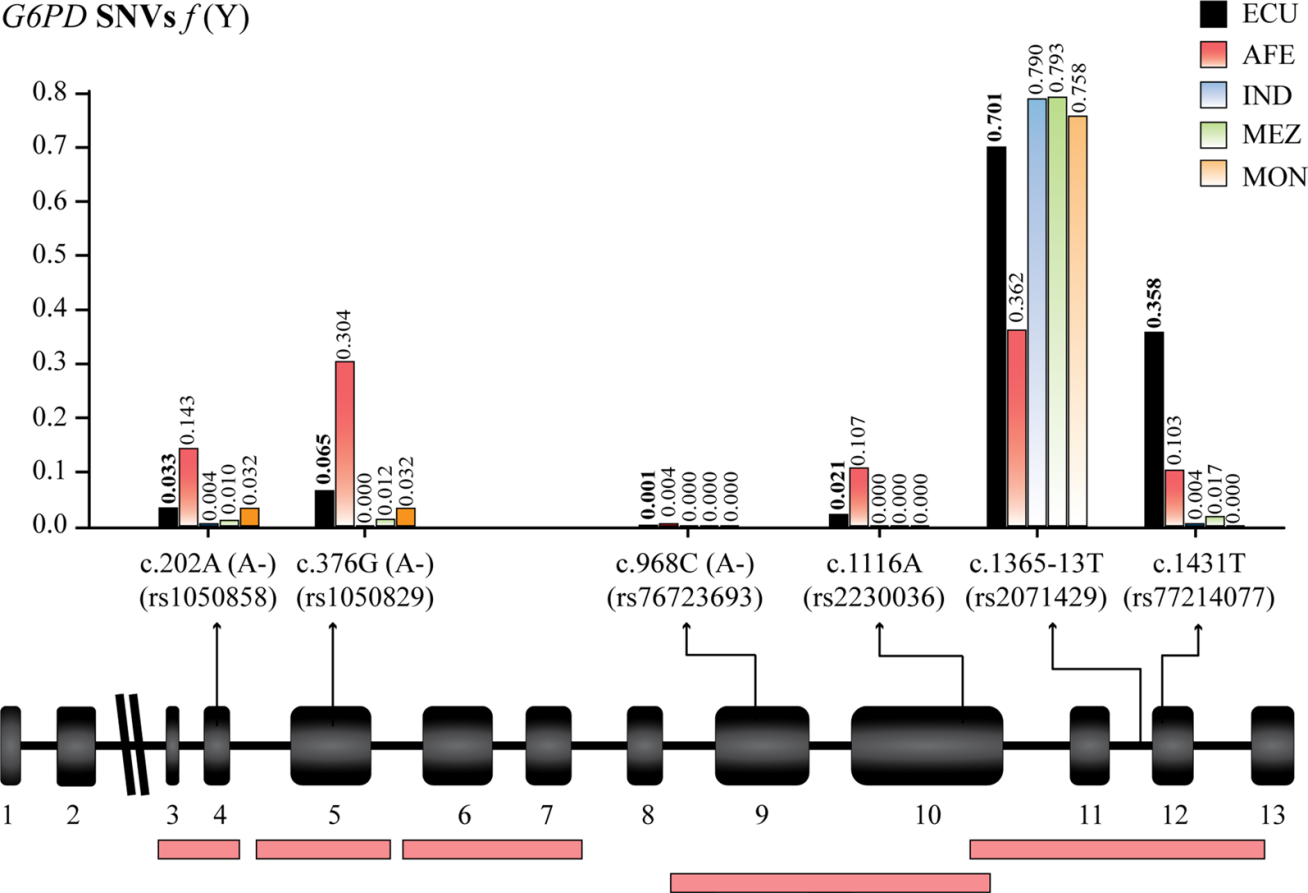
Schematic representation of the *G6PD* gene and the pharmacogenetic variants screened in the Ecuadorian population. The schematic diagram shows the exonic (black boxes) and intronic regions of the *G6PD* gene. The amplified regions are indicated with boxes in pink (at the bottom of the figure). The stacked bar chart shows the allele frequencies for the minor allele of the total Ecuadorian population (ECU) and for the Afro-Ecuadorian (AFE), Indigenous (IND), Mestizo (MEZ), and Montubio (MON) ethnic groups

The class III A- c.202G > A and class IV A- c.376 A > G variants are the most prevalent in the AFE groups when compared to other *G6PD* variants in the total population. The MAFs in AFE from the HG (Imbabura and Carchi), and the CO (Esmeraldas) reach 0.143 and 0.304 respectively. On the other hand, only one AFE heterozygous individual was recorded in the province of Esmeraldas for the SNV A- c.968T > C (rs76723693). Additionally, a high prevalence was observed for the intronic variant c.1365-13 C > T (rs2071429) in the Ecuadorian population (MAF = 0.701). Allele frequencies of 0.358 and 0.021 are recorded for the minor allele of variants class IV c.1116G > A (rs2230036) and c.1431 C > T (rs77214077) respectively (Fig. 1).

### Hardy Weinberg equilibrium, pairwise genetic distances and AMOVA

The HWE analysis for the Ecuadorian female population shows statistical differences for loci A- c.202G > A, A- c.968T > C, c.1116G > A, and c.1365-13 C > T (p < 0.001). However, these differences disappear when considering ethnic self-determination and when developing the analysis by AFE, IND, MEZ, and MON groups. No statistically significant deviations from HWE expectations were detected for the 6 loci in the studied population when considering the groups by ethnic self-determinations and geographic location (Additional file 1: Table S3).

**Fig. 2:**
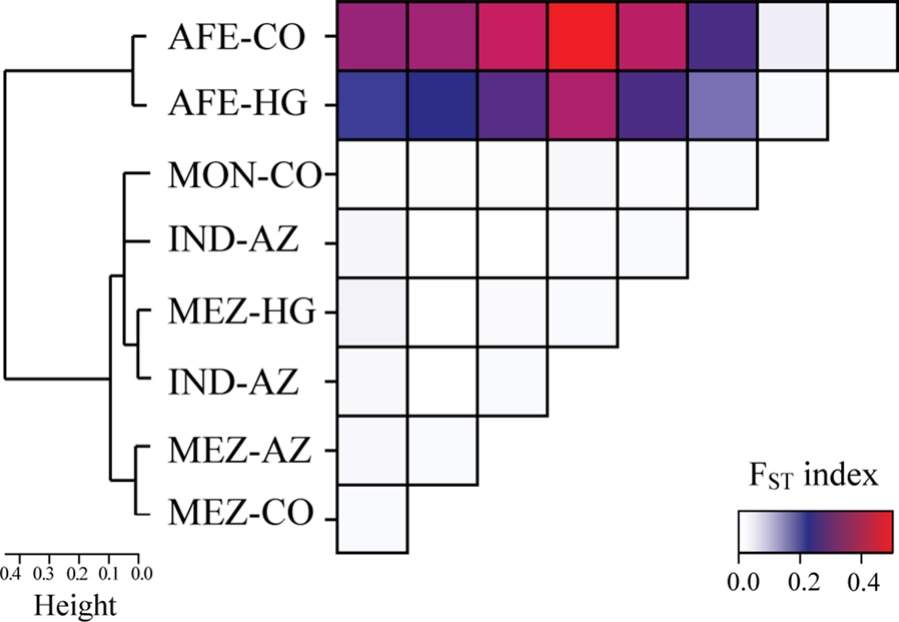
Genetic structure of the Ecuadorian ethnic groups evaluated by pairwise F_ST_ analyses. Dendrogram and heat map generated from F_ST_ genetic distances. F_ST_ values were calculated from SNVs found in the *G6PD* gene. The four Ecuadorian ethnic groups (Afro-Ecuadorian: AFE; Indigenous: IND; Mestizo: MEZ; and Montubio: MON) and their geographic distribution (Coast: CO; Highlands: HG; and Amazon Region: AZ) are considered

The pairwise analysis shows a high degree of differentiation between Ecuadorian ethnic populations when calculating F_ST_ indices, especially between AFE and each of the three other populations: IND (F_ST_ = 0.222, p = < 0.0001), MEZ (F_ST_ = 0.278, p = < 0.0001) and MON (F_ST_ = 0.172, p = < 0.0001). When considering the distribution of ethnic groups by geographic region, there is a certain degree of differentiation between MEZ populations in the HG and those in the CO (F_ST_ = 0.0145, p = 0.04505) (Additional file 1: Table S4). The high degree of divergence between AFE and the other Ecuadorian ethnic groups is evident in the dendrogram generated (Fig. 2). This differentiation was confirmed by applying an AMOVA between the ethnic groups studied. The calculated variability between AFE, MEZ, IND, and MON groups is 19.16, while within geographic groups it is 80.01.

### Prevalence of G6PD deficiency in ecuadorian ethnic groups

The genotyping results for the Ecuadorian population show the presence of class III A- c.202 A and A- c.968 C, and class IV A- c.376G alleles. The A- c.376G allele by itself is not considered a variant that induces a deficiency in the enzymatic activity of G6PD, but in association with A- c.202 A or A- c.968 C it leads to the deficient enzyme. Furthermore, heterozygous females who carry the minor allele can be regarded as deficient patients due to epigenetic X-chromosome inactivation [34]. Due to the recessive X-linked inheritance of the *G6PD* gene and in order to estimate population deficiency by provinces, the calculation incorporated both hemizygous men for A- c.202 A and heterozygous and homozygous women for the A- c.202 A and A- c.968 C alleles. The calculated prevalence of G6PDd in the general Ecuadorian population is 0.046. However, when analysing the distribution by ethnic self-identification, it is observed that the AFE population presents a higher frequency (0.192) than the IND groups (0.014). For MEZ and MON populations, individuals with diminished phenotypes are not recorded (Additional file 1: Table S5).

### Spatial distribution of malaria cases and G6PD deficiency in Ecuador

**Fig. 3:**
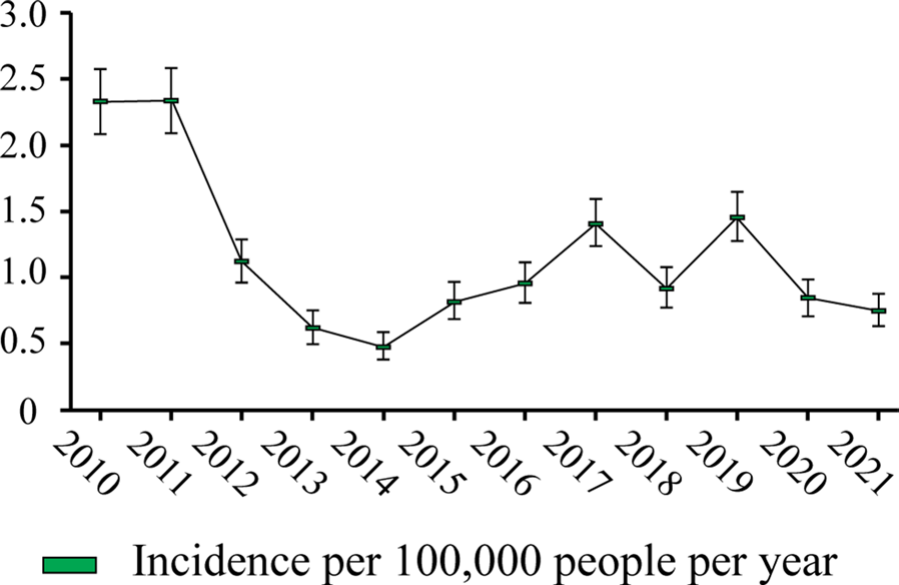
Incidence of hospitalized cases of malaria in Ecuador during the years 2010–2021

From 2010 to 2021, 2,277 malaria hospitalized cases were reported in Ecuador. 1,351 were males and 925 were females. The average annual incidence rate was 1.16 cases per 100,000 inhabitants ([1.11;1.21] 95% Poisson confidence intervals). The yearly evolution of hospital records showed a decline in the incidence of malaria through the years (Fig. 3). Poisson regression indicated a negative significant association of malaria cases with recent years (p < 0.00001).

**Fig. 4:**
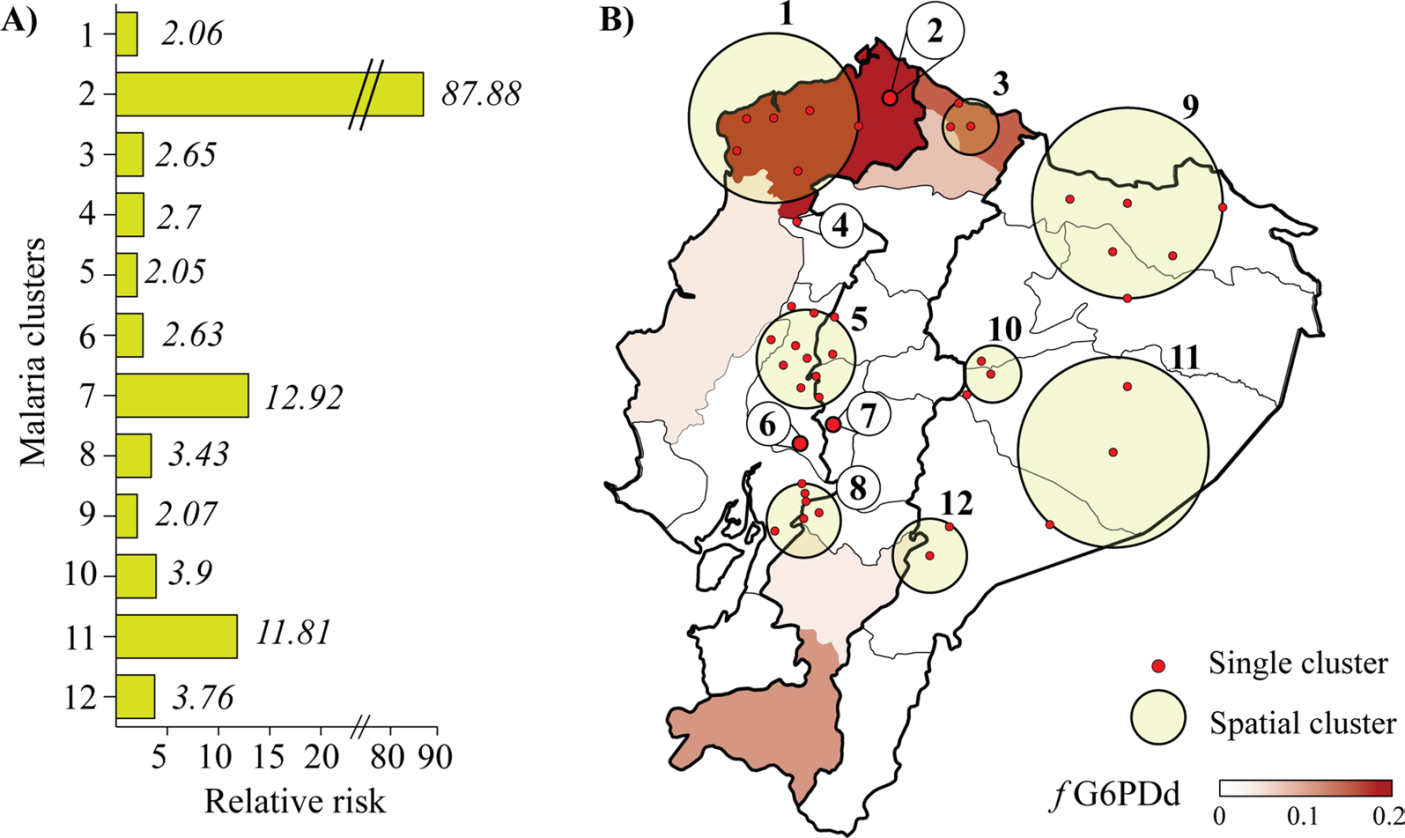
Spatial distribution of malaria cases and G6PD deficiency in Ecuador. **A** Relative risk for malaria clusters identified in Ecuador. **B** Spatial distribution of malaria clusters and frequency of G6PD deficiency in the Ecuadorian population

From the reported hospitalized cases, a total of twelve significant clusters of malaria infection in Ecuador were identified, 4 of these as single spots and 8 as spatial clusters (Additional file 1: Table S6). The clusters with the highest relative risk rates (RR) are identified as single spots in the CO region, specifically the provinces of Esmeraldas (RR = 87.88, p = 0.0001) and Los Ríos (RR = 12.92, p = 0.0001), and as a spatial cluster in AZ, namely the provinces of Pastaza and Morona Santiago (RR = 11.81, p = 0.0001). The remaining clusters exhibit similar relative risk rates, ranging from 2.05 to 3.09 (Fig. 4A).

As shown in Fig. 4B, the spatial clusters primarily concentrate in the centre-north CO and AZ regions. By comparing the distribution of these clusters with the calculated prevalence of G6PDd for the entire population, notable observations can be made. Specifically, the clusters located in the provinces of Esmeraldas (clusters 1 and 11) and Carchi (cluster 8) are situated in geographic regions where a prevalence of G6PD deficiency is evident in genotyped individuals. In contrast, the clusters identified in AZ (clusters 3, 4, 10, and 12) and CO-HG region (clusters 2, 4, 5, 6, 7, and 9) are located in provinces with low prevalence of G6PD deficiency.

## Discussion

MAFs for the SNVs in *G6PD* show differences across the groups studied. The HWE analysis reveals statistical differences for the A- c.376 A > G, A- c.968T > C, c.1116G > A, and c.1365-13 C > T loci in the total population. However, these disparities disappeared upon analysis by ethnic group, suggesting a potential substructure [35]. This divergence is corroborated by clustering analysis and calculated F_ST_ values (Fig. 2). In particular, the AFE group exhibits a high genetic divergence compared with the NAM, MEZ, and MON groups. Nonetheless, no significant differences were observed between the AFE subgroups of CO and HG regions. These results align with previous findings on pharmacogenetic variants in the Ecuadorian population [36], indicating that genetic variability in the *G6PD* allows a genetic characterization of the different ethnicities present in Ecuador.

G6PDd is one of the most common inherited disorders, affecting approximately 400 million people worldwide, representing 4.87% of the global population [37]. This deficiency shows significant variation in its prevalence, being almost nonexistent in the original Amerindian populations and reaching 20% or more in parts of Africa and Asia [38]. In addition, the distribution of variants associated with a decrease in G6PD enzymatic activity presents a distinctive pattern in populations worldwide. The A- c.202G > A and A- c.968T > C variants are predominant in Afro-descendant groups, whereas the Mediterranean *G6PD* variant c.563 C > T is predominant in West Asia [21]. In this study, it was identified the presence of class IV A- c.376 A > G and class III A- c.202G > A and A- c.968T > C variants, associated with moderate and severe deficiency, respectively. As reported, the class III A- c.202 A allele is the most common in Latin American groups, and together with A- c.968 C, it represents the genetic basis of the deficiency in more than 90% of individuals throughout the region [21, 22, 24]. These variants are predominantly found in AFEs, aligning with the global distribution where Afro-descendant populations exhibit higher frequencies of A- c.202G > A and A- c.968T > C variants compared to European, Asian, and Indigenous North and South American populations [15, 39]. The presence of these pharmacogenetic markers in this group may be attributable to the transatlantic slave trade from West African populations. Conversely, the absence of European *G6PD*d variants in the studied population is consistent with its genetic basis, especially considering that the European ancestral genetic contribution is not predominant in the general population [40–43].

The main objective of this study is to determine the prevalence of G6PDd in different Ecuadorian ethnic groups, which together with the identification of malaria clusters, can facilitate the implementation of effective treatment strategies and significantly reduce the likelihood of toxicity associated with the use of anti-malarial therapies. From 2006 to 2018, a total of 9,429 cases of *Plasmodium* sp. were documented in Ecuador [44]. Malaria-endemic regions are primarily located along the Pacific coast in western Ecuador, the inter-Andean valleys in central Ecuador, and the Amazon River basin in the eastern part of the country [45]. This distribution is consistent with the spatial clusters identified in this study. Specific provinces highlighted as major geographic foci of infection, such as Esmeraldas, Imbabura, Carchi, and AZ provinces, provide valuable information for targeted surveillance, prevention, and control efforts. This knowledge enables the allocation of resources to areas of greatest need, including improved access to diagnostic, treatment, and vector control measures.

It is noteworthy that Ecuador, like many South American countries excluding Venezuela, has witnessed a significant decrease in malaria prevalence in recent years, and most cases are now reported as asymptomatic [22, 46]. The study found a significant reduction in malaria incidence, as evidenced by hospital records over the years (Fig. 3). While this decline is a positive development for the population, it presents a challenge, as untreated cases in endemic regions act as crucial reservoirs for ongoing disease transmission. Although occasional outbreaks of *P. falciparum* are reported in Ecuador [47], the primary cause of malaria cases can be attributed to *P. vivax*. Specifically, along the northern coast of Ecuador, *P. vivax* accounts for 97.9% of infections, with *P. falciparum* accounting for 2.1% [26]. A similar pattern is observed in the Lower Napo region, where *P. vivax* is the dominant parasite, responsible for 92% of reported cases [48]. Considering this distribution of *Plasmodium* species, it is crucial to implement effective pharmacological strategies. The recommended primary prophylaxis strategy against *P. vivax* involves the use of primaquine, which is also recommended as a first-line treatment in eliminating *P. vivax* hypnozoites and *P. falciparum* gametocytes [49–52]. According to the Ministry of Public Health of Ecuador [53], the recommended protocol for malaria treatment includes the utilization of 8-aminoquinoline drugs, with a specific focus on primaquine as a complementary therapeutic agent. In cases of non-complicated malaria caused by *P. falciparum*, the primary treatment entails TCA (artemether + lumefantrine), coupled with a single administration of primaquine at a dosage of 0.75 mg per kilogram of body weight. In cases of non-complicated malaria due to *P. vivax* or *Plasmodium ovale*, the treatment strategy consists of 3-day administration of chloroquine accompanied by a 14-day regimen of primaquine at a dosage of 0.5 mg. It is important to exercise caution with chloroquine and hydroxychloroquine due to their potential anti-malarial efficacy in inducing oxidative stress through the generation of haem-based reactive oxygen species [20, 54]. Individuals with G6PDd should be especially careful as they are at an elevated risk of adverse effects [55, 56].

In Ecuador, different ethnic groups show a specific spatial distribution due to historical and social events, which places special value on the present study. The effects of G6PDd in malaria-exposed populations of different ethnic origins can be characterized with less bias than in other regions where ethnic groups are mixed, or where there is dynamic variation among them. As shown in Fig. 4B, the deficiency calculated by the alleles A- c.202 A and A- c.968 C is geographically and ethnically limited, and is concentrated in the north of the country, specifically in Esmeraldas (CO region) along with Imbabura and Carchi (HG region). In contrast, the IND groups within the AZ do not exhibit markers associated with G6PDd. Considering the spatial relationship found between malaria foci and G6PDd distribution, treatment strategies should be tailored to AFE groups distributed in the northern region of the country.

The complex interplay between genetics, ethnicity, and disease susceptibility, shaped by historical and societal influences on malaria distribution and genetic factors such as G6PDd, offers invaluable insights. Understanding these dynamics can guide the development of culturally sensitive strategies for malaria prevention and control, considering the diverse needs and vulnerabilities of different ethnic groups. Patient management requires careful consideration of the individual risk/benefit ratio in determining the most suitable treatment approach. In order to mitigate the occurrence of adverse clinical outcomes associated with anti-malarial drugs, it is essential to know the patient’s G6PD status prior to prescribing any 8-aminoquinoline-based drug [57]. For example, a safer alternative for treating identified G6PDd-positive cases could involve administrating primaquine at a dose of 0.75 mg/kg/week for a period of 8 weeks. Considering the relationship found between foci of infection and deficiency in the north of the country, it is vital to develop and implement screening methods to ensure the safe and effective use of anti-malarial drugs, thus minimizing the potential risks of drug-induced toxicity [58, 59]. By incorporating genetic screening into the treatment approach, healthcare professionals can tailor treatment plans to improve patient safety and optimize therapeutic outcomes.

This study provides for the first time the prevalence and distribution of genetic variants associated with G6PDd within the Ecuadorian population, encompassing its four primary ethnic groups. However, there are inherent limitations. The sample size of 581 individuals might not capture the full genetic diversity of the population, suggesting that a larger sample could offer a more comprehensive insight into G6PDd and its relation to malaria patterns in Ecuador. Similarly, the employed methodology facilitated a genetic screening for prevalent variants in G6PDd that hold predictive value in malaria treatment. While several coding regions of the gene were screened (9 out of 13 exons), the need remains for forthcoming studies to employ high-throughput sequencing techniques and broaden the coverage of genotyping. Lastly, the spatial cluster analysis relied on publicly available data, which might be prone to reporting biases or data gaps, further exacerbated by the COVID-19 pandemic. Although the calculated incidence rate shows a decreasing trend in all the years analysed, the possible biases introduced during this pandemic period highlight the need for a cautious interpretation of the findings.

## Conclusions

The genotyping analysis of the *G6PD* gene identified the presence of class III A- c.202G > A (rs1050828) and A- C.968T > C (rs76723693) variants, both of which are associated with a reduction in G6PD enzymatic activity. The frequency of these variants differed between the studied Ecuadorian ethnicities, with the AFE groups showing the highest prevalence. Evaluating G6PDd has significant implications for the population in question, especially in light of the spatial distribution of reported malaria cases in Ecuador and the distribution of G6PDd molecular variants. Twelve clusters as endemic malaria areas in Ecuador were identified, mainly distributed along the northern coast (Esmeraldas), in the Andean region (Carchi), and the Amazon region (Sucumbíos, Orellana, Pastaza, and Morona Santiago). Given the high prevalence of G6PDd in AFE groups (19.2%) located in the northern (both the coastal and the Andean region) of the country, coupled with the elevated rates of malaria infection identified in this geographic region (a relative risk ranging from 2.06 to 87.88), there is a pressing need to implement genetic screening strategies within these ethnic communities. This would mitigate toxicity risks when administrating anti-malarial therapies. The integration of genetic screening into patient management enables health professionals to optimize treatment plans, improve patient safety, and enhance therapeutic outcomes.

### Supplementary Information

**Additional file 1:** **Table S1. **Primer sequences and amplification conditions for genotyped regions in *G6PD*. **Table S2. **Allelic and genotypic frequencies of SNVs in *G6PD* found in different Ecuadorian ethnic groups. **Table S3. **Observed and expected values of heterozygotes and significant p-values calculated for the Hardy-Weinberg equilibrium of molecular variants of *G6PD* in Ecuadorian females. **Table S4. **Pairwise genetic distances (F_ST_) calculated from the *G6PD *genetic variants between the Ecuadorian populations grouped by geographic regions. **Table S5. **G6PDd in different Ecuadorian ethnic groups. **Table S6. **Spatial analysis of significant malaria clusters across Ecuador.

## Data Availability

The datasets used during the current study are available from the corresponding author on reasonable request.

## Abbreviations

AFE: Afro-Ecuadorian
AZ: Amazonia
AMOVA: Analysis of molecular variance
CO: Coast
G6PD: Glucose-6-phosphate dehydrogenase
G6PDd: G6PD deficiency
HG: Andes
HWE: Hardy-Weinberg equilibrium
IND: Indigenous
MAF: Minor allele frequency
MEZ: Mestizo
MON: Montubio
NADPH: nicotinamide adenine dinucleotide phosphate
RR: Relative risk rate
SNV: Single Nucleotide Variant
